# Lactational Serotonergic Perturbation Imprints Stress-Related Transcriptional Profiles in the Adolescent Female Rat Prefrontal Cortex

**DOI:** 10.1021/acschemneuro.6c00082

**Published:** 2026-05-22

**Authors:** Maria Teresa Gallo, Aman Miezan Emmanuel Acquah, Luca Sbabo, Fabio Fumagalli, Paola Brivio, Francesca Calabrese

**Affiliations:** Department of Pharmacological and Biomolecular Sciences “Rodolfo Paoletti”, Università degli Studi di Milano, Milan 20133, Italy

**Keywords:** serotonin, adolescence, behavior, female rats, mood disorders

## Abstract

Early life perturbations of serotonin (5-hydroxytryptamine, 5-HT) signaling induce long-term neurobehavioral consequences. We previously demonstrated that perinatal manipulation of the 5-HTergic system in rats leads to distinct adult biobehavioral outcomes depending on sex and time of intervention, with postnatal manipulations leading to cognitive deficits in females. Moreover, we showed that prenatal disruption of 5-HT signaling alters the response to acute stress in male adolescents. Here, we investigated whether postnatal exposure to the selective serotonin reuptake inhibitor fluoxetine (FLX) affects adolescent behavior and molecular stress responsivity. Rats were exposed to FLX during the lactation period and behaviorally characterized during adolescence to assess distinct psychiatric-like phenotypes. Transcriptional responses to acute restraint stress (ARS) were evaluated in the prefrontal cortex (PFC) and the dorsal and ventral hippocampus. Although no behavioral alterations were detected, postnatal FLX exposure modified the ARS-induced molecular response. In particular, females exposed to FLX during lactation showed a blunted induction of immediate early genes, early response genes, and brain-derived neurotrophic factor isoforms in the PFC. These findings indicate an altered stress-responsive transcriptional profile that may represent an early molecular alteration preceding the cognitive deficits previously observed selectively in adult females.

## Graphical abstract

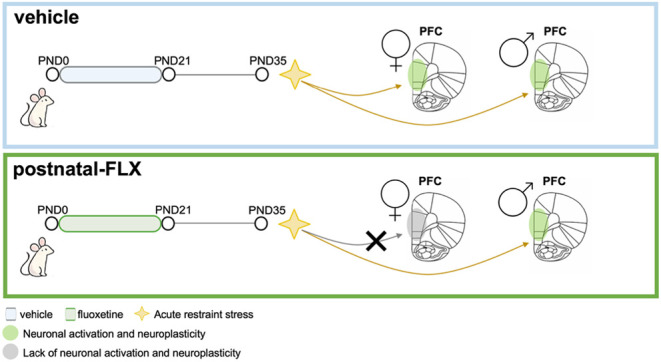

## Introduction

Serotonin (5-hydroxytryptamine, 5-HT) plays a fundamental role in brain development by regulating key processes such as neuronal proliferation, migration, synaptogenesis, and circuit refinement.^1–5^ Perturbations of 5-HTergic signaling during early life have been shown, in preclinical models, to induce long-lasting alterations in brain function and behavior.^2,6–11^ In line with these observations, clinical evidence suggests that altered 5-HTergic neurotransmission during infancy and adolescence contributes to the etiology of psychiatric disorders.^12–15^

Supporting this notion, our recent studies employing a pharmacological manipulation of 5-HT in early life, through administration of the selective serotonin reuptake inhibitor fluoxetine (FLX) in rats, demonstrated that developmental disruption of the 5-HT signaling leads to enduring behavioral outcomes that depend on sex and the timing of the pharmacological manipulation.^16,17^ Specifically, males were more sensitive to prenatal FLX exposure, exhibiting an anhedonic-like phenotype, whereas females were more sensitive to postnatal FLX exposure, displaying cognitive deficits, only in adulthood but not earlier.

Beyond its developmental role, 5-HT is also a key modulator of the stress response.^18–23^ Accordingly, we previously observed that male rats prenatally exposed to FLX exhibit an altered response to acute restraint stress (ARS) during adolescence, preceding the emergence of the pathological-like phenotype.^24^

In the present study, we focused on the postnatal period, precisely throughout the whole lactation, to test whether manipulation of the serotonergic system early after birth shapes the adolescent response to a subsequent environmental challenge in terms of neuronal activation. Moreover, by including both male and female animals, we also investigated potential FLX-induced differences in the response to an acute stress in each sex. Specifically, after the behavioral characterization, we performed an expanded transcriptional profiling to investigate molecular mechanisms associated with stress response. In particular, we analyzed multiple components of the activity-dependent transcriptional cascade triggered by acute stress, assessing the expression of immediate early genes (IEGs: *Arc*, *cFos*, and *Zif268*), and early response genes (ERGs: *Nr4a1, Npas4, Dusp1, Sgk1*, and *Gadd45β*), which are rapidly induced by acute stimuli and widely used as markers of neuronal activity.^25–33^ In parallel, given the importance of plasticity in the appropriate adaptation to acute stressors, we measured the expression of *Bdnf* and its main isoforms, given its critical role in regulating synaptic plasticity and stress-related neuroadaptations.^34–41^

These analyses were conducted in key cortical and limbic regions involved in stress processing and emotional regulation, including the prefrontal cortex (PFC) and the dorsal and ventral subfields of the hippocampus (dHip and vHip, respectively). These regions were selected because they play distinct and region-specific roles in the regulation of stress responsivity, with the PFC critically involved in the control of stress responses and the hippocampal subfields contributing differentially to cognitive and emotional aspects of stress processing.

By focusing on postnatal FLX exposure and evaluating female and male rats separately, this work adds a new dimension to understanding how the timing of 5-HTergic perturbations shapes neurobehavioral trajectories across development.

## Results and Discussion

### Postnatal FLX Administration Does Not Affect Behavior in Adolescent Male and Female Rats

As shown in Figure 1A,B, following postnatal exposure to FLX, both female and male rats underwent the sucrose preference test (SPT), open field (OF) test, and novel object recognition (NOR) test during adolescence. Statistical analyses revealed no significant differences in sucrose preference (%) (Figure 1C,D), time spent in the center of the OF arena (Figure 1E,F), number of entries into the center, or distance traveled in the center (Supporting Information, Table 3), nor in the NOR index (%) (Figure 1G,H). Furthermore, the behavioral composite score (BCS), an index integrating the outcomes of the single behavioral assessments (Supporting Information, Table 2), revealed no significant differences between FLX-exposed female (Figure 1I) and male (Figure 1J) rats compared to their respective controls. These results are consistent with our previous findings^16,17^ and indicate that the pathological-like phenotype induced by postnatal FLX exposure emerges at adulthood (see refs 16,17 ), and not at earlier developmental stages.

**Figure 1:**
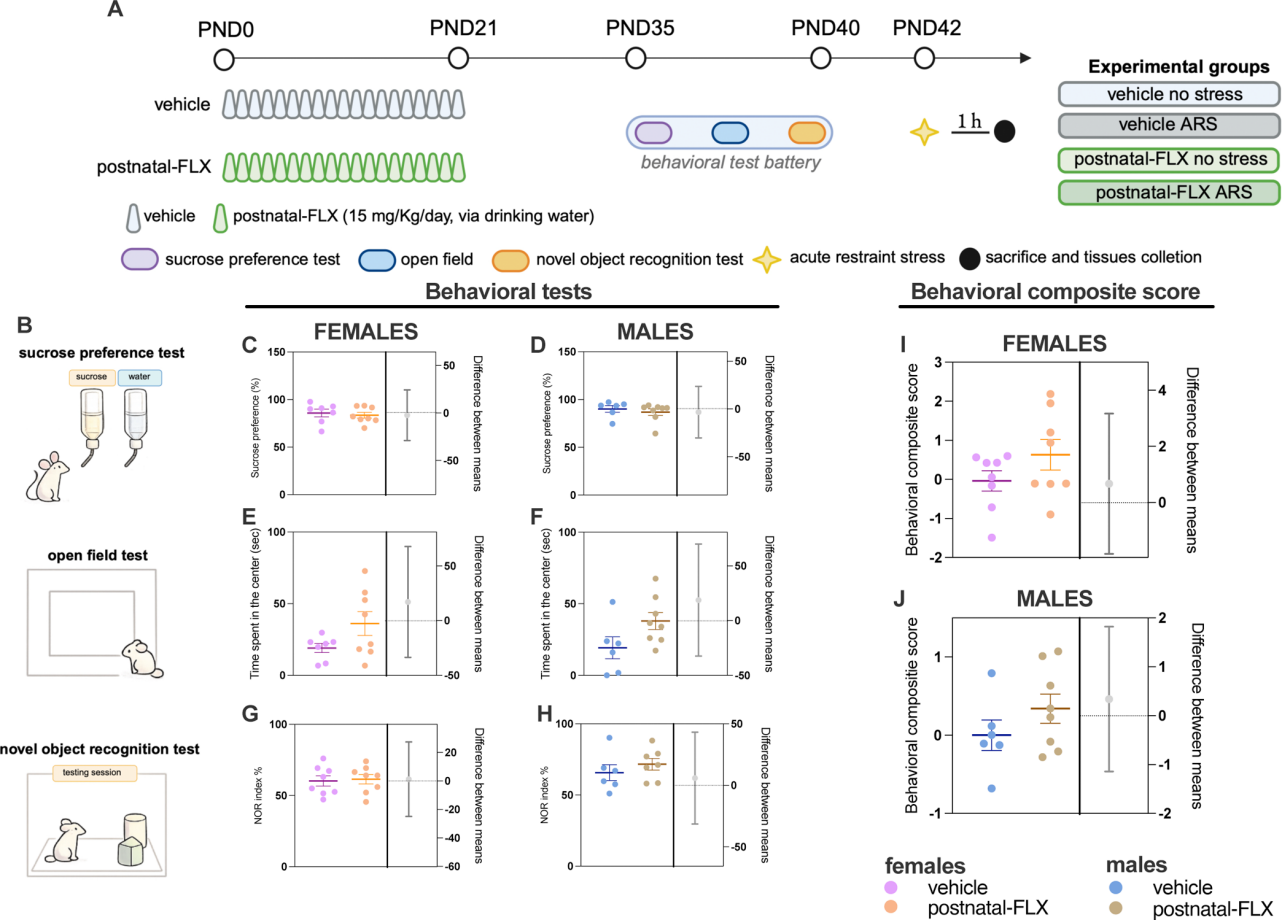
Experimental design and behavioral assessment in adolescent female and male rats exposed to postnatal fluoxetine. Panel (A): Female and male Wistar rats were exposed to FLX (15 mg/kg/day, via drinking water) or vehicle (water) during lactation. During adolescence, animals underwent a behavioral test battery: the sucrose preference test, the open field test, and the novel object recognition test. At PND 42, rats were subjected to 1 h of ARS and sacrificed 1 h later. Panel (B): Schematic representation of the behavioral test battery. Panels (C, D): Sucrose preference % of female (C) and male (D) rats. Panels (E, F): Time spent in the center of the OF arena by female (E) and male (F) rats. Panels (G, H) NOR index % of female (G) and male (H) rats. Panels (I, J): Behavioral composite score (BCS) in female (C) and male (D) rats (*n* = 6–8 per group). Data are presented as estimation plots showing individual data points and group means ± SEM on the left axis, and the effect size expressed as the difference between means on the right axis. The precision of the effect size estimate is indicated by the 95% confidence interval. An unpaired *t-*test was performed. PND: postnatal day, FLX: fluoxetine, ARS: Acute restraint stress.

### Postnatal FLX Administration Inhibits the ARS-Induced Neuronal Activation in Adolescent Female Rats

To map the neuronal activation in the PFC, dHip, and vHip in response to ARS, we assessed the expression of the IEGs *Arc, cFos*, and *Zif268* as well as the ERGs *Nr4a1, Npas4, Dusp1, Sgk1*, and *Gadd45β*. For each brain region, we calculated a *z*-activation score to provide an integrated measure of gene modulation following ARS (Figure 2A–C). Distinct patterns of activation emerged depending on sex and brain region.

**Figure 2:**
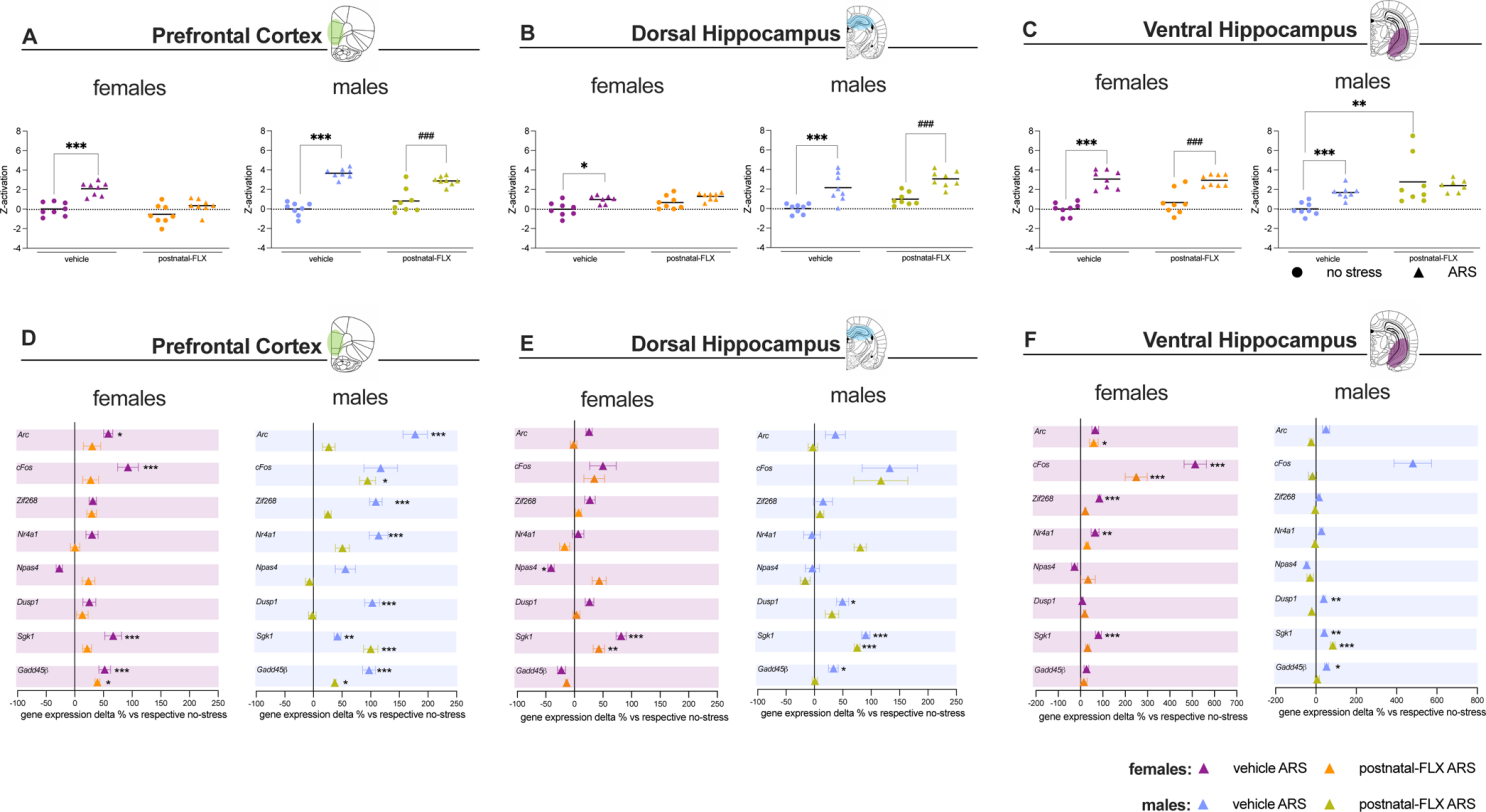
z-Activation scores and mRNA expression analyses in the prefrontal cortex (PFC), dorsal hippocampus (dHip), and ventral hippocampus (vHip) of female and male rats exposed to postnatal fluoxetine (FLX) and acute restraint stress (ARS) in adolescence. Panels A–C: *z*-activation in the PFC (A), dHip (B), and vHip (C) of female (left) and male (right) rats (*n* = 8 per group). Data are expressed as the mean with individual data points. *p < 0.05, **p < 0.01, ***p < 0.001 vs vehicle no-stress group; ^###^ *p* < 0.001 vs postnatal-FLX no-stress group (two-way ANOVA followed by Tukey’s multiple comparisons test). Panels (D–F): ARS-induced changes in mRNA expression of *Arc, cFos, Zif268, Nr4a1, Npas4, Dusp1, Sgk1*, and *Gadd45β* genes in the PFC (D), dHip (E), and vHip (F) of female (left) and male (right) rats, expressed as the percentage change relative to their respective no-stress controls. Data are expressed as the mean ± SEM; *p < 0.05, **p < 0.01, ***p < 0.001 vs respective no-stress group (two-way ANOVA followed by Tukey’s multiple comparison tests). FLX: fluoxetine, ARS: Acute restraint stress.

Indeed, perturbation of the 5-HTergic system during lactation mainly affected the stress response in the PFC and the dHip of female rats, whereas in the same regions of males, a comparable ARS-induced activation was observed in both vehicle and postnatal-FLX groups.

Accordingly, in female rats (Figure 2A,B), we found significant effects of postnatal FLX exposure (PFC: *F*_1,31_ = 17.30, *p* < 0.001; dHip: *F*_1,30_ = 5.847, *p* < 0.05; two-way ANOVA), ARS (PFC: *F*_1,31_ = 29.85, *p* < 0.001; dHip: *F*_1,30_ = 15.33, *p* < 0.001; two-way ANOVA) and of their Interaction (PFC: *F*_1,31_ = 4.738, *p* < 0.05; two-way ANOVA).

Post hoc Tukey’s multiple comparisons test showed a significant increase in *z*-activation following ARS in vehicle females (PFC: *p* < 0.001; dHip: *p* < 0.05; vs vehicle no stress), whereas no ARS-induced changes were observed in the postnatal-FLX animals.

In contrast, in males, we detected a significant effect of ARS in PFC and dHip (PFC: *F*_1,31_ = 104.8, *p* < 0.001; dHip: *F*_1,31_ = 41.48, *p* < 0.001; two-way ANOVA). Indeed, Tukey’s post hoc analysis revealed increased *z*-activation following ARS in vehicle as well as in postnatal-FLX males (vehicle ARS: *p* < 0.001, postnatal-FLX ARS: *p* < 0.001 vs respective no-stress controls) in both brain regions.

Differently, in the vHip (Figure 2C) of female rats, two-way ANOVA highlighted a significant effect only of ARS (*F*_1,31_ = 69.98, *p* < 0.001), and the post hoc analysis showed an increase in *z*-activation following ARS in both vehicle (*p* < 0.001 vs vehicle no stress) and postnatal-FLX (*p* < 0.001 vs postnatal-FLX no stress) female rats. In contrast, in males, we did not find any significant effect of ARS (see Supporting Information, Table 4, for complete data and statistics).

By examining the expression of the individual genes contributing to the *z*-activation score (Figure 2D–F), we were able to better determine which genes drive the observed effects.

In the PFC (Figure 2D) of female rats, ARS induced a significant upregulation of multiple genes selectively in the vehicle group (*Arc*: + 58% *p* < 0.05; *cFos*: + 93% *p* < 0.001; *Sgk1*: + 67% *p* < 0.001; *Gadd45β:* + 52% *p* < 0.001 vs vehicle no stress).

Differently, in males, we found an overall increase in the IEGs measured in both the experimental groups (see Supporting Information, Tables 6–11, for complete data and statistics).

In the dHip (Figure 2E), ARS induced a trend toward an upregulation of most genes selectively in the vehicle group, while *Sgk1* expression was significantly upregulated following ARS in both females (vehicle ARS: + 82%, *p* < 0.001, postnatal-FLX ARS + 43%, *p* < 0.01 vs respective no stress controls) and males (vehicle ARS: + 91%, *p* < 0.001, postnatal-FLX ARS + 75%, *p* < 0.001 vs respective no stress controls) regardless of early life FLX exposure.

Moreover, in males, ARS increased *Dusp1* (+50% *p* < 0.05 vs vehicle no stress) and *Gadd45β* (+33% *p* < 0.05 vs vehicle no stress) mRNA levels selectively in the vehicle group.

In the vHip (Figure 2F) of female rats, statistical analysis revealed ARS-induced increase in *cFos* (+514% *p* < 0.001 vs vehicle no stress), *Zif268* (+84% *p* < 0.001 vs vehicle no stress), *Nr4a1* (+65% *p* < 0.01 vs vehicle no stress), and *Sgk1* (+80% *p* < 0.001 vs vehicle no stress) expression in vehicle females, while only *cFos* (+250% *p* < 0.001 vs postnatal-FLX no stress) and *Arc* (+60% *p* < 0.05 vs postnatal-FLX no stress) were upregulated in postnatal-FLX females.

Differently, in the vHip of male rats, ARS induced *Sgk1* expression in both vehicle (+41% *p* < 0.01 vs vehicle no stress) and postnatal-FLX (+83% *p* < 0.001 vs postnatal-FLX no stress) animals. Furthermore, *Dusp1* (+39% *p* < 0.01 vs vehicle no stress) and *Gadd45β* (+53% *p* < 0.05 vs vehicle no stress) were significantly upregulated following ARS exclusively in vehicle males.

These results indicate that postnatal FLX exposure alters the transcriptional response to acute stress by blunting the rapid induction of IEGs and ERGs. While ARS activated stress-related transcriptional programs in vehicle-exposed animals, this response was attenuated in postnatal-FLX rats, particularly in females. The absence of ARS-induced increases in the z-activation score in the PFC and dHip of the postnatal-FLX group points to an impaired ability to engage IEGs and ERGs expression following an acute challenge, suggesting a reduced capacity to recruit plasticity-related mechanisms in response to stress. Particularly, ARS-induced upregulation of key activity-dependent genes, including *Arc*, *cFos*, *Dusp1*, and *Gadd45β*, was largely confined to vehicle animals and attenuated or absent in postnatal-FLX rats. Notably, *Arc* and *cFos* play a central role in activity-dependent plasticity,^42–45^ linking neuronal activation to adaptive changes in synaptic function and memory-related processes. In this context, the parallel between the altered transcriptional response and the cognitive deficits observed in adult female postnatal-FLX rats^16,17^ is noteworthy and suggests a possible link between disrupted stress-induced gene activation and long-term cognitive vulnerability.

Of note, similar alterations in stress-evoked transcriptional responses have been consistently reported in our previous studies across different animal models of 5-HTergic manipulation. Specifically, we demonstrated that ARS-induced upregulation of *Arc* is blunted in 5-HT transporter knockout rats,^46^ Tph1 knockout rats^21^ and Tph2 knockout rats.^47^ Likewise, the lack of ARS-induced *cFos* upregulation observed in postnatal-FLX rats is in line with our earlier findings in Tph1 and Tph2 knockout models.^21,47^ Along the same line, ARS-induced upregulation of *Dusp1* and *Gadd45β* was also found to be affected by postnatal-FLX, consistent with our previous observations in Tph1 knockout rats and in a chronic stress model of depression.^48^

In contrast, *Sgk1* appears to represent a reliable marker of acute stress responsiveness that is only modestly influenced by 5-HTergic alterations, in line with our previous studies.^21,24,49^

The convergence of these findings across pharmacological and genetic approaches supports the notion that proper 5-HTergic signaling during early life is required for the normal recruitment of activity-dependent gene programs in response to stress later in life.

### Postnatal FLX Administration Blunted ARS-Induced *Bdnf* Upregulation in the PFC of Adolescent Female Rats

The expression of the neurotrophin *Bdnf* was measured in the PFC, dHip, and vHip as a marker of neuroplasticity, given its well-established role in the acute stress response. Specifically, we quantified the mRNA levels of total *Bdnf*, *Bdnf* long 3′ UTR, *Bdnf* isoform IV, and *Bdnf* isoform VI because they represent functionally distinct *Bdnf* isoforms involved in dendritic mRNA localization, activity-dependent transcriptional regulation, antidepressant function, and long-term synaptic and stress-related adaptations.^50–54^ For each brain region, a *Bdnf* z-score was calculated to provide an integrated measure of neuroplasticity changes following the ARS exposure.

ARS induced a physiological increase in the *Bdnf* z-score selectively in the PFC (Figure 3A,D), whereas no changes were detected in either the dHip (Figure 3B,E) or the vHip (Figure 3C,F) (see Supporting Information, Table 5, for Complete Data and Statistics). Notably, postnatal FLX exposure altered the ARS-induced response specifically in female rats, while no comparable effect was observed in male rats.

**Figure 3:**
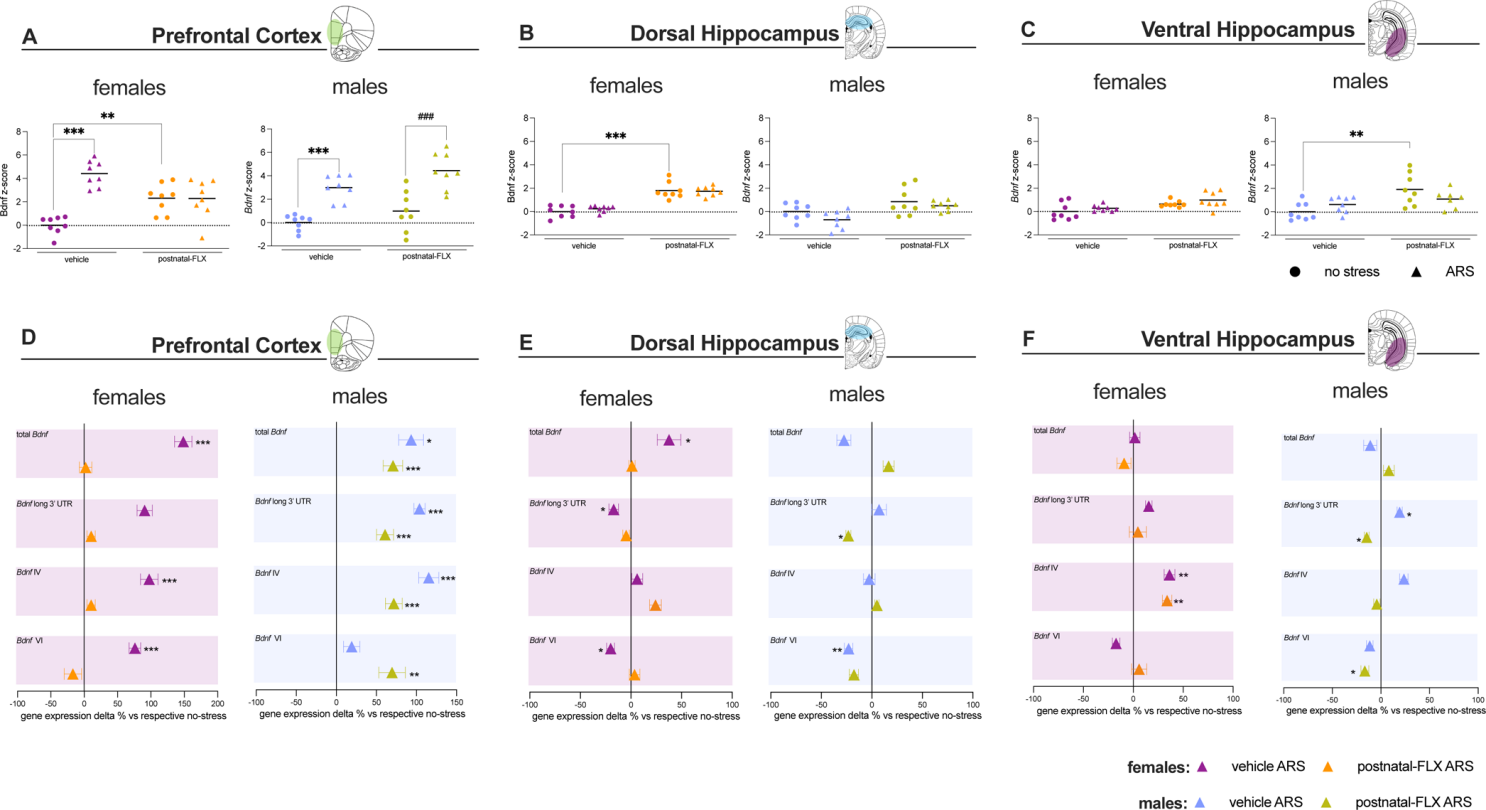
Bdnf ***z-*** score and mRNA expression analyses in the prefrontal cortex (PFC), dorsal hippocampus (dHip), and ventral hippocampus (vHip) of female and male rats exposed to postnatal fluoxetine (FLX) and acute restraint stress (ARS) in adolescence. Panels (A–C): *Bdnf* *z*-score in the PFC (A), dHip (B), and vHip (C) of female (left) and male (right) rats (*n* = 8 per group). Data are expressed as the mean with individual data points. **p < 0.01, ***p < 0.001 vs vehicle no-stress group; ^###^ *p* < 0.001 vs postnatal-FLX no-stress group (two-way ANOVA followed by Tukey’s multiple comparisons test). Panels (D–F): ARS-induced changes in mRNA expression of total *Bdnf, Bdnf* long 3′UTR, *Bdnf* isoform IV, and *Bdnf Isoform VI* genes in the PFC (D), dHip (E), and vHip (F) of female (left) and male (right) rats, expressed as the percentage change relative to their respective no-stress controls. Data are expressed as the mean ± SEM; *p < 0.05, **p < 0.01, ***p < 0.001 vs respective no stress group (two-way ANOVA followed by Tukey’s multiple comparisons test). FLX: fluoxetine, ARS: Acute restraint stress.

Indeed, in the PFC of female rats (Figure 3A), two-way ANOVA revealed significant effects of ARS (*F*_1,31_ = 28.08, *p* < 0.0001). Two-way ANOVA and the interaction between postnatal FLX and ARS (*F*_1,31_ = 25.71, *p* < 0.0001, two-way ANOVA). Tukey’s post hoc analysis showed a significant ARS-induced increase in the *Bdnf* z-score in the vehicle females (*p* < 0.001), an effect that was blunted by postnatal-FLX exposure.

In contrast, in males, we observed significant effects of both FLX (*F*_1,31_ = 6.912, *p* < 0.05; two-way ANOVA) and ARS (*F*_1,31_ = 48.71, *p* < 0.001; two-Way ANOVA). In particular, the multiple comparison analyses revealed an upregulation of the *Bdnf* z-score regardless of the postnatal exposure to FLX (vehicle ARS: *p* < 0.001; postnatal-FLX ARS: *p* < 0.001 vs respective no stress controls).

According to the region and sex differences, in the PFC, the Tukey’s multiple comparisons test highlighted an ARS-induced upregulation of total *Bdnf* (+148% *p* < 0.001), *Bdnf* isoform IV (+97% *p* < 0.001), and *Bdnf* isoform VI (+76%*p* < 0.001) selectively in the vehicle females, whereas postnatal FLX blunted these ARS-induced increases.

By contrast, in males, we observed an overall ARS-induced upregulation of the different *Bdnf* isoforms in both vehicle and postnatal-FLX groups (see Supporting Information, Tables 6–11, for complete data and statistics).

These findings indicate that postnatal FLX exposure compromises the ability to mount an appropriate neuroplastic response to acute stress, as reflected by the lack of transcriptional activation of *Bdnf* isoforms. Under physiological conditions, ARS increases total *Bdnf* as well as isoforms IV and VI, consistent with the well-established role of this neurotrophin in stress-induced synaptic plasticity.^55,56^ However, in postnatally FLX-exposed animals, this adaptive response is absent, suggesting an impairment of activity-dependent transcriptional mechanisms. Given the close bidirectional interaction between 5-HTergic signaling and BDNF-dependent plasticity, early life disruption of 5-HT may have long-lasting consequences on the ability to recruit BDNF-mediated adaptive responses to ARS.^57,58^ Importantly, the failure to activate *Bdnf* expression aligns with our previous results showing similar deficits in *Tph1* knockout rats^21^ and in a chronic stress model of depression.^59^ Moreover, we have demonstrated that pre- and postnatal FLX exposure, as well as SERT deletion, induce long-lasting alterations in neuroplasticity-related pathways.^17,60^ Altogether, these data support the hypothesis that postnatal FLX exposure induces persistent changes in the 5-HTergic network, ultimately leading to reduced neuroplastic flexibility and an impaired ability to adapt to acute stress later in life.

Here, we confirm that postnatal FLX exposure does not induce detectable behavioral alterations during adolescence in either male or female rats, in line with our previous evidence showing that the same manipulation leads to cognitive deficits only in adulthood, particularly in females.^16,17^ Despite the absence of behavioral changes during adolescence, postnatal FLX exposure profoundly altered the molecular response to an acute challenge, thereby unmasking dysfunctions at the transcriptional level. Specifically, the transcriptional response to ARS was blunted or absent in FLX-exposed female rats. Notably, a similar outcome was reported in our previous study showing that adolescent male rats exposed to FLX during gestation failed to support an appropriate molecular response to an acute stress, even before the emergence of the pathological-like phenotype.^24^

Together, these findings reinforce the idea that perinatal 5-HTergic alterations disrupt developmental trajectories and compromise stress responsivity at the molecular level well before the onset of behavioral abnormalities. Our results further support the notion of a different susceptibility between males and females to 5-HTergic perturbations in early life. This sex bias likely reflects fundamental differences in how 5-HTergic signaling contributes to neurodevelopment, as sexual brain dimorphism emerges early in life due to differential exposure to gonadal hormones, particularly the surge of testosterone during fetal and early postnatal stages in males, which shapes sex-specific trajectories of brain maturation.^61,62^ In addition, sex differences in the rate of basal brain 5-HT synthesis suggest that the 5-HTergic tone per sé may influence vulnerability to developmental perturbations^63^ in both sexes. In this context, the role of 5-HT in the maturation of the hypothalamic–pituitary–gonadal axis,^64^ as well as the different timing of its maturation across sexes, may further contribute to these sex-specific sensitivities. Within this context, female rats exhibited the most pronounced deficits within the PFC, a key hub for stress integration and regulation.^65^ In controls, ARS elicited an increase in IEGs, ERGs, and *Bdnf* isoforms transcripts, reflecting an adaptive neuroplastic response. This effect was almost abolished by postnatal FLX exposure, indicating an impairment in the ability of the PFC to translate acute stress signals into appropriate molecular responses. The greater vulnerability of the PFC may reflect the heightened sensitivity of this region to 5-HTergic manipulation during the first stages of development, consistent with the evidence that 5-HT plays a crucial role in shaping prefrontal maturation and plasticity.^66,67^ Notably, it cannot be excluded that postnatal FLX exposure alters the temporal dynamics of the stress response, potentially leading to a delayed or anticipated transcriptional activation following ARS, an aspect that warrants further investigation. It is also important to consider that, since IEGs and ERGs are expressed in multiple neuronal populations, FLX exposure could affect potential cellular and receptor targets of these players in diverse cell types. 5-HTergic signaling via 5-HT1A, 5-HT2A, and 5-HT3 receptors, which are differentially expressed in the developing male and female PFC, may mediate these effects, influencing neuronal maturation, synaptic plasticity, and circuit refinement.^68–73^ In addition, FLX may exert effects on BDNF/TrkB signaling,^74,75^ potentially altering activity-dependent neurotrophic support and synaptic maturation. Dissecting receptor-specific mechanisms and cell-type contributions will be crucial to fully understand how early life FLX exposure programs long-lasting molecular and circuit-level changes.

Finally, beyond central 5-HTergic mechanisms, more than 90% of peripheral 5-HT is produced in the gastrointestinal tract. FLX, by inhibiting the serotonin transporter peripherally, may alter gut 5-HT availability, modulating vagal afferent signaling and systemic immune mediators that influence central stress-responsive circuits. Through these gut–brain pathways, peripheral 5-HTergic levels could indirectly modulate central ones and, in turn, regulate the IEGs and ERGs expression following acute stress, suggesting that both central and peripheral 5-HT systems may shape the long-term impact of early life FLX exposure.

This study has some limitations that must be acknowledged. First, although changes in immediate early gene and early response gene expression after acute stress are widely used as reliable markers of neuronal activation, the inclusion of protein level analyses would have provided a more comprehensive characterization of the molecular response. Future studies integrating transcriptomic and proteomic approaches will help strengthen these findings.

Second, we tested only a single dose of FLX. This dose was intentionally selected to ensure consistency with our previous work and to allow cross-study comparability. Importantly, it falls within the range commonly used to experimentally manipulate the serotonergic system in preclinical models, rather than to mimic therapeutic dosage. While a dose–response analysis would certainly add further insight, our experimental design was aimed at isolating mechanistic effects under controlled conditions.

Finally, maternal behavior during lactation was not directly assessed. Although alterations in maternal care following FLX exposure cannot be excluded and could indirectly influence offspring development and stress responsiveness, our primary objective was to investigate direct neurobiological outcomes in the offspring. Future studies specifically addressing maternal behavior will be important to disentangle direct pharmacological effects from potential indirect environmental influences.

In conclusion, our findings reveal that postnatal FLX exposure induces a dissociation between behavioral and molecular phenotypes during adolescence. While behavior appears to be unaffected at this developmental stage, acute stress unmasks profound alterations in stress-responsive transcriptional and neuroplastic mechanisms, particularly in the PFC of females. These molecular dysfunctions likely represent a latent vulnerability that predisposes individuals to the emergence of cognitive deficits in adulthood. Overall, this study supports a model in which early life 5-HTergic perturbation programs long-lasting alterations in stress-related molecular pathways, setting the stage for delayed, rather than immediate, psychiatric disorder manifestation.

## Materials and Methods

### Animals

One month prior to the start of the experiment, eight adult female and eight adult male Wistar rats (Charles River, Germany) were acclimated to the animal facility. Each female was subsequently mated with a male and monitored twice daily for the presence of a vaginal plug. The day of plug detection was defined as the gestational day 0. Following confirmation of mating, males were returned to their original housing, whereas pregnant females were housed individually and were provided with nesting material.

Offspring were weaned at postnatal day (PND) 21, separated by sex, and housed in groups of two to four animals per cage. A total of 64 animals (32 females and 32 males) originating from eight dams were used and allocated to experimental groups of eight animals each (see Supporting Information, Table 1).

Animals had ad libitum access to food and water and were maintained under controlled environmental conditions, including a 12 h light/dark cycle, ambient temperature of 22 ± 2 °C, and relative humidity of 50 ± 5%.

All procedures performed in this study were conducted in accordance with authorization no. 472/2021-PR approved by the Italian Health Ministry with the Italian legislation in animal experimentation (DL 26/2014) and conformed to the European Communities Council Directive of September 2010 (2010/63/EU).

All procedures were conducted with the aim of minimizing animal distress and reducing the number of animals employed, and the study was performed in accordance with ARRIVE guidelines.

### Experimental Paradigm and Drug Administration

Following mating, dams were randomly allocated to the different experimental groups. Specifically, four dams were assigned to the postnatal-FLX group and exposed to the selective serotonin reuptake inhibitor FLX throughout the lactation period, beginning on PND0 until PND21, whereas four dams were assigned to the control group and received the vehicle only (water). As in our previous studies,^16,17,24^ FLX was administered by dissolving the compound directly in the drinking water, resulting in an estimated intake of 15 mg/kg/day. An excess volume of the solution was prepared to accommodate daily water consumption. To ensure accurate dosing, baseline water intake was monitored prior to the initiation of FLX administration to determine the appropriate dilution. Throughout the exposure period, the solution was freshly prepared each day, and the drug concentration was adjusted according to the dams’ daily water intake and body weight. This administration protocol was designed to account for physiological changes in body weight and fluid consumption occurring during the lactation period.

As shown in Figure 1A, upon completion of the pharmacological manipulation, animals were left undisturbed in their home cages until the onset of the stress procedure.

### Behavioral Tests

The behavioral test battery was performed in adolescent offspring, following a predefined schedule that included SPT, OF, and NOR tests (Figure 1A). The sequence of assessments was intentionally arranged from the least to the most invasive to minimize the potential carryover effects of earlier procedures on subsequent behavioral outcomes. All behavioral assessments were performed by an experimenter who was blind to the experimental group allocation.

#### Sucrose Preference Test

As previously described,^16^ animals underwent a three-day habituation period in their home cages, during which they were trained to drink from two bottles. Following habituation, rats were subjected to 15 h of food and water deprivation and then housed individually. During the 1 h testing phase, each animal was given access to two bottles, one containing a 1% sucrose solution and the other containing water. To avoid spatial bias, the sucrose solution was placed in the position that had been identified as less preferred during the habituation period. Sucrose preference was calculated as follows: [(mL of sucrose solution consumed)/(total mL of fluid consumed)] × 100.

#### Open Field Test

Animals were tested in a nontransparent square open field arena (50 × 50 × 40 cm). Animals were placed individually in the center of the arena and allowed to freely explore the apparatus for 5 min. Time spent in the center area of the arena was used as indices of anxiety-like behavior and it was quantified, as well as the number of entries and the distance traveled in the center, using a combination of automated tracking (ANY-maze software, Ireland) and manual scoring.

#### Novel Object Recognition Test

Animals were tested in a nontransparent square open field arena (50 × 50 × 40 cm). As previously reported,^76^ the protocol consisted of three phases: a 5 min training session with two identical plastic bottles, a 1 h intertrial interval in the home cage, and a 5 min test session with one familiar object (plastic bottle) and one novel object (tin can). Exploration time was quantified using a combination of automated tracking (ANY-maze software, Ireland) and manual scoring. The NOR index was calculated as [(time spent exploring the novel object)/(total time spent exploring both objects)] × 100.

### Acute Restraint Stress

Two days after completion of the behavioral test battery, animals from each experimental group were randomly subdivided into stressed (ARS) and nonstress subgroups. Rats assigned to the stress condition were subjected to a single 1 h session of ARS by placement in flexible, transparent plastic cones with an opening at the bottom. Restraint was performed between 9:30 AM and 12:45 PM to minimize potential confounding effects of circadian rhythms.

The final experimental groups were as follows: vehicle no stress, vehicle ARS, postnatal FLX no stress, and postnatal FLX ARS (8 per sex per group). For details on group subdivision and litter information, see Supporting Information, Table 1.

### Tissue Collection

One hour after the end of the ARS, animals were decapitated. The PFC, dHip, and vHip were dissected from the whole brain, frozen on dry ice, and stored at −80 °C for later analyses. Precisely, the PFC corresponds to plates 6–13, dHip to 43–72, and vHip to 73–84 according to the atlas of Paxinos and Watson.^77^

### RNA Preparation and Gene Expression Analysis by Quantitative Real-Time PCR

Total RNA was isolated from the frozen brain tissues by a single step of guanidinium isothiocyanate/phenol extraction using PureZol RNA isolation reagent (Bio-Rad Laboratories, Segrate, Italy) and quantified by spectrophotometric analysis as previously described.^78^ To avoid DNA contamination, samples were treated with DNase (ThermoFisher Scientific, Italy).

Real-time polymerase chain reaction (q-PCR) was performed to assess *Arc, c-Fos, Nr4a1, Zif268, Npas4, Dusp1, Sgk1, Gadd45β*, Total *Bdnf*, *Bdnf* long 3′ UTR, *Bdnf* isoform IV, and *Bdnf* isoform VI mRNA levels.

Primer sequences used were purchased from Eurofins MWG-Operon (Supporting Information, Table 12) and Life Technologies (Supporting Information, Table 13).

RNA was analyzed by a TaqMan qRT-PCR instrument (CFX384 real-time system, Bio-Rad Laboratories, Italy) using the iScriptTM one-step RT-PCR kit for probes (Bio-Rad Laboratories, Segrate, Italy) (see ref 79 for details). Samples were run in 384-well formats in triplicate as multiplexed reactions with the normalizing internal control 36b4.

### Behavioral Composite Score, *z-*Activation, and *Bdnf z*-Score

Behavioral data were standardized using *z*-score normalization $\left(z=\frac{X-\mu }{\sigma }\right)$ , where *X* represents the individual value for each animal and μ and σ correspond to the mean and standard deviation of the vehicle group, respectively. The BCS was calculated by first converting the outcome of each behavioral parameter into a *z*-score and then averaging the standardized values for each animal. Specifically, the BCS (Figure 1I,J) integrated sucrose preference % in the SPT, time spent in the center of the arena during the OF test, and the NOR Index % from the NOR test.

Similarly, gene expression data were standardized using the same z-score normalization, with μ and σ defined as the mean and standard deviation of the vehicle no stress group. Z-activation (Figure 2A–C) was calculated as the average z-score of the IEGs and ERGs, while *Bdnf* z-score was obtained by averaging the z-score of the individual *Bdnf* isoform examined (Figure 3A–C).

### Statistical Analysis

GraphPad Prism software version 10.4 (GraphPad Software Inc., CA, USA) was employed for the analysis of all of the results. The behavioral results were analyzed with the unpaired *t*-test, and the data are presented in estimation plots (Figure 1). The molecular results were subjected to a two-way analysis of variance (ANOVA), with postnatal-FLX and ARS as variables, followed by the Tukey’s multiple comparison tests. Statistical significance for all of the tests was assumed for *p* < 0.05.

## Supplementary Material



## Notes

Complete data and statistical analyses of behavioral tests, mRNA analyses, z-scores, a summary of the number of animals used in the experiment, and the primers and probes used in qPCR assessment (PDF)
