# Supplementary material for: Lactational Serotonergic Perturbation Imprints Stress-Related Transcriptional Profiles in the Adolescent Female Rat Prefrontal Cortex
Source: ACS Chem Neurosci. 2026 May 22;17(11):2122–31. doi: 10.1021/acschemneuro.6c00082 (PMC13237732; doi:10.1021/acschemneuro.6c00082)
Supplement: Supplementary file 1 [file cn6c00082_si_001.pdf]

Lactational serotonergic perturbation imprints stress-related transcriptional profiles in the adolescent female rat prefrontal cortex

Maria Teresa Gallo[1], Aman Miezan Emmanuel Acquah[1], Luca Sbabo[1], Fabio Fumagalli[1], Paola Brivio[1]*, Francesca Calabrese[1].

[1]Department of Pharmacological and Biomolecular Sciences “Rodolfo Paoletti”, Università degli Studi di Milano, Milan, Italy

***Corresponding author:** Dr Paola Brivio

Department of Pharmacological and Biomolecular Sciences

Università di Milano, Via Balzaretti 9, 20133 Milan, Italy

Phone: +39-02 50318332

E-mail: paola.brivio@unimi.it

ORCID ID: orcid.org/0000-0003-1837-6519

SUPPLEMENTARY TABLES

EXPERIMENTAL GROUP	LITTER	n° FEMALES no stress	n° FEMALES ARS	n° MALES no stress	n° MALES ARS
**vehicle**	1	3		2	
	2	1	2		2
	3		2	3	3
	4	4	4	3	3
	**tot:**	**8**	**8**	**8**	**8**
**postnatal-FLX**	5	2	2	2	2
	6	2	2	2	2
	7	4		4	2
	8		4		2
	**tot:**	**8**	**8**	**8**	**8**
	**tot:**	**16**	**16**	**16**	**16**

Supplementary Table 1: Number of rats used in the experiment divided into the experimental groups.

	Behavioral result	Unpaired *t*-test	vehicle	postnatal-FLX
**females**	SP %	p=0.6462	86±4	84±3
	NOR index %	p=0.82015	60±4	61±3
	Time spent in the center (sec)	p=0.0930	19±8	32±5
**males**	SP %	p=0.5260	90±3	87±3
	NOR index %	p=0.4095	66±6	72±4
	Time spent in the center (sec)	p=0.0706	19±3	36±8

Supplementary Table 2. Behavioral test results in **adolescent female and male rats** exposed to postnatal-FLX. Data are expressed as mean ± SEM of independent measures. Unpaired *t*-test.

	Behavioral parameter	Unpaired *t*-test	vehicle	postnatal-FLX
**females**	Entries in the center	p=0.9523	19±6	18±2
	Distance travelled in the center (m)	p=0.5635	3.1±1	2.8±0
**males**	Entries in the center	p=0.5322	13±5	16±2
	Distance travelled in the center (m)	p=0.3436	18±1	2.5±0

Supplementary Table 3. Open field test results in **adolescent female and male rats** exposed to postnatal-FLX. Data are expressed as mean ± SEM of independent measures. Unpaired *t*-test.

sex	Brain region	Two-way ANOVA	F (dFn, dFd)	p value	*z*-activation: Tukey's multiple comparisons test			
					Vehicle no stress	Vehicle ARS	postnatal -FLX no stress	postnatal-FLX ARS
females	PFC	FLX	F (1,31) = 17.30	p=0.0003	0.013	2.090 ***	1.147	-0.526
		ARS	F (1,31) = 29.85	p<0.0001				
		FLX X ARS	F (1,31) = 4.738	p=0.0381				
	dHip	FLX	F (1,30) = 5.847	p=0.8299	-0.019	0.979 *	0.670	1.290
		ARS	F (1,30) = 15.33	p=0.0006				
		FLX X ARS	F (1,30) = 0.8299	p=0.3704				
	vHip	FLX	F (1,31) = 0.7362	p=0.3982	0.000	3.069 ***	0.674	2.943 $^{\#\#\#}$
		ARS	F (1,31) = 69.98	p<0.0001				
		FLX X ARS	F (1,31) = 1.570	p=0.2206				
males	PFC	FLX	F (1,31) = 0.003617	p=0.9525	-0.003	3.662 ***	0.817	2.875 $^{\#\#\#}$
		ARS	F (1,31) = 104.8	p<0.0001				
		FLX X ARS	F (1,31) = 8.254	p=0.0077				
	dHip	FLX	F (1,31) = 8.164	p=0.0080	-0.002	2.133 ***	0.964	3.032 $^{\#\#\#}$
		ARS	F (1,31) = 41.48	p<0.0001				
		FLX X ARS	F (1,31) = 0.01066	p=0.9185				
	vHip	FLX	F (1,30) = 11.90	p=0.0019	0.000	1.688	2.770 **	2.402
		ARS	F (1,30) = 1.696	p=0.2038				
		FLX X ARS	F (1,30) = 4.137	p=0.0519				

Supplementary Table 4: Statistical analysis of z-activation in the **prefrontal cortex (PFC), dorsal hippocampus (dHip) and ventral hippocampus (vHip) of female and male rats** exposed to postnatal FLX and subjected to acute restraint stress (ARS) during adolescence. Data are expressed as mean ± SEM of independent measures. $^{*}p<0.05$, $^{**}p<0.01$, $^{***}p<0.001$ vs vehicle no stress; $^{\#\#\#}$ $p<0.001$ vs postnatal-FLX no stress; two-way ANOVA with Tukey's multiple comparison test.

sex	Brain region	Two-way ANOVA	F (dFn, dFd)	p value	*Bdnf* *z*-score: Tukey's multiple comparisons test			
					Vehicle no stress	Vehicle ARS	postnatal -FLX no stress	postnatal-FLX ARS
females	PFC	FLX	F (1,31) = 0.03935	p=0.8442	0.000	4.410 ***	2.777 **	2.306
		ARS	F (1,31) = 25.08	p<0.0001				
		FLX X ARS	F (1,31) = 25.71	p<0.0001				
	dHip	FLX	F (1,31) = 88.13	p<0.0001	0.000	0.240	1.795 ***	1.734
		ARS	F (1,31) = 0.2605	p=0.6138				
		FLX X ARS	F (1,31) = 0.7333	p=0.3991				
	vHip	FLX	F (1,31) = 12.78	p=0.0013	0.000	0.278	0.649	0.987
		ARS	F (1,31) = 2.628	p=0.1162				
		FLX X ARS	F (1,31) = 0.02514	p=0.8752				
males	PFC	FLX	F (1,31) = 6.912	p=0.0137	0.000	2.991 ***	0.983	4.450 $^{\#\#\#}$
		ARS	F (1,31) = 48.71	p<0.0001				
		FLX X ARS	F (1,31) = 0.2793	p=0.6013				
	dHip	FLX	F (1,31) = 12.46	p=0.0015	0.000	-0.692	0.844	0.493
		ARS	F (1,31) = 3.302	p=0.0799				

		FLX X ARS	F (1,31) = 0.3515	p=0.5581				
	vHip	FLX	F (1,30) = 12.25	p=0.0016	0.000	0.623	1.915 ***	1.084
		ARS	F (1,30) = 0.09302	p=0.7627				
		FLX X ARS	F (1,30) = 4.586	p=0.0414				

Supplementary Table 5: Statistical analysis of *Bdnf* z-score in the **prefrontal cortex (PFC), dorsal hippocampus (dHip) and ventral hippocampus (vHip) of female and male rats** exposed to postnatal FLX and subjected to acute restraint stress (ARS) during adolescence. Data are expressed as mean ± SEM of independent measures. **p<0.01, ***p<0.001 vs vehicle no stress; ### p<0.001 vs postnatal-FLX no stress; two-way ANOVA with Tukey's multiple comparison test.

Gene	Two-way ANOVA	F (dFn, dFd)	p value	Tukey's multiple comparisons test			
				Vehicle no stress	Vehicle ARS	postnatal-FLX no stress	postnatal-FLX ARS
Arc	FLX	F (1,31) = 0.9172	p=0.3464	100± 8	158±8 *	123±12	159±19
	ARS	F (1,31) = 14.95	p=0.0006				
	FLX X ARS	F (1,31) = 0.7508	p=0.3936				
cFos	FLX	F (1,31) = 7.828	p=0.0092	100±12	193±18 ***	92±16	117±13
	ARS	F (1,31) = 15.54	p=0.0005				
	FLX X ARS	F (1,31) = 5.085	p=0.0321				
Zif268	FLX	F (1,31) = 1.953	p=0.1733	100±19	131±6	87±7	113±8
	ARS	F (1,31) = 6.417	p=0.0172				
	FLX X ARS	F (1,31) = 0.06847	p=0.7955				
Nr4a1	FLX	F (1,30) = 5.812	p=0.0230	100±7	130±10	94±8	95±8
	ARS	F (1,30) = 3.171	p=0.0862				
	FLX X ARS	F (1,30) = 2.916	p=0.0992				
Npas4	FLX	F (1,31) = 26.46	p<0.0001	100±12	73±5	42±4 ***	52±5
	ARS	F (1,31) = 1.174	p=0.2879				
	FLX X ARS	F (1,31) = 5.529	p=0.0260				
Dusp1	FLX	F (1,31) = 2.400	p=0.1325	100±4	125±11	93±10	105±9
	ARS	F (1,31) = 4.286	p=0.0478				
	FLX X ARS	F (1,31) = 0.5083	p=0.4818				
Sgk1	FLX	F (1,31) = 25.14	p<0.0001	100±4	167±15 ***	83±3	100±6
	ARS	F (1,31) = 25.59	p<0.0001				
	FLX X ARS	F (1,31) = 8.835	p=0.0060				
Gadd45β	FLX	F (1,31) = 9.134	p=0.0053	100±9	152±10 ***	86±7	120±4 #
	ARS	F (1,31) = 31.06	p<0.0001				
	FLX X ARS	F (1,31) = 1.446	p=0.2393				
Total *Bdnf*	FLX	F (1,31) = 5.625	p=0.0248	100± 15	248±13 ***	208±12 ***	212±19
	ARS	F (1,31) = 26.07	p<0.0001				
	FLX X ARS	F (1,31) = 23.02	p<0.0001				
Bdnf long 3' UTR	FLX	F (1,31) = 1.04	p=0.3167	100±5	191±11 ***	130±10	143±8
	ARS	F (1,31) = 34.5	p<0.0001				
	FLX X ARS	F (1,31) = 19.1	p=0.0002				
Bdnf IV	FLX	F (1,31) = 1.254	p=0.2723	100±8	197±13 ***	134±11	137±14
	ARS	F (1,31) = 18.42	p=0.0002				
	FLX X ARS	F (1,31) = 16.54	p=0.0004				
Bdnf VI	FLX	F (1,30) = 5.770	p=0.0234	100±8	176±9 ***	168±12 **	140±22

	ARS	F (1,30) = 9.742	p=0.0043
	FLX X ARS	F (1,30) = 16.37	p=0.0004

Supplementary Table 6: Statistical analysis of *Arc*, *cFos, Zif268*, *Nr4a1*, *Npas4, Dusp1, Sgk1*, *Gadd45*β, total *Bdnf*, *Bdnf* long 3'UTR, *Bdnf* isoform IV *Bdnf* isoform VI mRNA levels in the **prefrontal cortex of female rats** exposed to postnatal FLX and subjected to acute restraint stress (ARS) during adolescence. Data are expressed as mean ± SEM of independent measures. *p<0.05, **p<0.01, ***p<0.001 vs vehicle no stress; # p<0.05 vs postnatal-FLX no stress; two-way ANOVA with Tukey's multiple comparison test.

Gene	Two-way ANOVA	F (dFn, dFd)	p value	Tukey's multiple comparisons test			
				Vehicle no stress	Vehicle ARS	postnatal-FLX no stress	postnatal-FLX ARS
Arc	FLX	F (1,30) = 0.7197	p=0.4037	100±13	278±21 ***	154±18	195±17
	ARS	F (1,30) = 29.07	p<0.0001				
	FLX X ARS	F (1,30) = 15.30	p=0.0006				
cFos	FLX	F (1,31) = 3.040	p=0.0922	100±23	218±29	144±45	280±20 #
	ARS	F (1,31) = 17.12	p=0.0003				
	FLX X ARS	F (1,31) = 0.09481	p=0.7604				
Zif268	FLX	F (1,30) = 0.07985	p=0.7797	100±8	209±11 ***	140±13	175±8
	ARS	F (1,30) = 48.39	p<0.0001				
	FLX X ARS	F (1,30) = 12.46	p=0.0015				
Nr4a1	FLX	F (1,31) = 5.751	p=0.0234	100±11	214±16 ***	96±19	145±12
	ARS	F (1,31) = 29.48	p<0.0001				
	FLX X ARS	F (1,31) = 4.652	p=0.0398				
Npas4	FLX	F (1,28) = 0.2516	p=0.6203	100±13	156±18	124±21	116±9
	ARS	F (1,28) = 2.261	p=0.1452				
	FLX X ARS	F (1,28) = 4.110	p=0.0534				
Dusp1	FLX	F (1,30) = 0.02790	p=0.8686	100±16	203±13 ***	151±16	148±10
	ARS	F (1,30) = 12.66	p=0.0014				
	FLX X ARS	F (1,30) = 14.27	p=0.0008				
Sgk1	FLX	F (1,31) = 3.668	p=0.0657	100±5	142±6 **	91±10	183±11 ###
	ARS	F (1,31) = 62.25	p<0.0001				
	FLX X ARS	F (1,31) = 8.618	p=0.0066				
Gadd45β	FLX	F (1,30) = 0.6082	p=0.4422	100±5	197±11 ***	118±18	162±2 #
	ARS	F (1,30) = 38.16	p<0.0001				
	FLX X ARS	F (1,30) = 5.459	p=0.0271				
Total *Bdnf*	FLX	F (1,30) = 22.05	p<0.0001	100± 8	193±15 *	179±28	305±22 ###
	ARS	F (1,30) = 29.25	p<0.0001				
	FLX X ARS	F (1,30) = 0.6682	p=0.4208				
Bdnf long 3' UTR	FLX	F (1,31) = 0.03095	p=0.8616	100±8	204±17 ***	118±14	189±12 ###
	ARS	F (1,31) = 67.79	p<0.0001				
	FLX X ARS	F (1,31) = 2.219	p=0.1475				
Bdnf IV	FLX	F (1,31) = 1.240	p=0.2749	100±7	215±13 ***	127±17	218±13 ###
	ARS	F (1,31) = 63.19	p<0.0001				
	FLX X ARS	F (1,31) = 0.8836	p=0.3553				
Bdnf VI	FLX	F (1,31) = 0.2809	p=0.6003	100±6	119±10	86±10	146±14 ##
	ARS	F (1,31) = 15.75	p=0.0005				

	FLX X ARS	F (1,31) = 3.650	p=0.0664

Supplementary Table 7: Statistical analysis of *Arc*, *cFos, Zif268*, *Nr4a1*, *Npas4, Dusp1, Sgk1*, *Gadd45*β β, total *Bdnf*, *Bdnf* long 3'UTR, *Bdnf* isoform IV *Bdnf* isoform VI mRNA levels in the **prefrontal cortex of male rats** exposed to postnatal FLX and subjected to acute restraint stress (ARS) during adolescence. Data are expressed as mean ± SEM of independent measures. **p<0.01, ***p<0.001 vs vehicle no stress; $^{\#\#}$p<0.01, $^{\#\#\#}$p<0.001 vs postnatal-FLX no stress; two-way ANOVA with Tukey's multiple comparison test.

Gene	Two-way ANOVA	F (dFn, dFd)	p value	Tukey's multiple comparisons test			
				Vehicle no stress	Vehicle ARS	postnatal-FLX no stress	postnatal-FLX ARS
Arc	FLX	F (1,31) = 0.03936	p=0.3464	100±13	126±5	112±11	110±7
	ARS	F (1,31) = 1.463	p=0.2366				
	FLX X ARS	F (1,31) = 1.929	p=0.1759				
cFos	FLX	F (1,29) = 15.10	p=0.0006	100±16	150±23	198±31	266±36
	ARS	F (1,29) = 4.626	p=0.0410				
	FLX X ARS	F (1,29) = 0.1142	p=0.7381				
Zif268	FLX	F (1,31) = 4.937	p=0.0345	100±8	127±9	127±8	136±6
	ARS	F (1,31) = 5.297	p=0.0290				
	FLX X ARS	F (1,31) = 1.297	p=0.2644				
Nr4a1	FLX	F (1,30) = 3.075	p=0.0909	100±13	107±10	137±13	113±12
	ARS	F (1,30) = 0.4781	p=0.4952				
	FLX X ARS	F (1,30) = 1.484	p=0.2337				
Npas4	FLX	F (1,30) = 2.037	p=0.1650	100±14	59±5 *	55±7 **	79±7
	ARS	F (1,30) = 0.9381	p=0.3414				
	FLX X ARS	F (1,30) = 13.95	p=0.0009				
Dusp1	FLX	F (1,31) = 0.02234	p=0.8823	100±10	126±8	113±10	117±7
	ARS	F (1,31) = 2.991	p=0.0947				
	FLX X ARS	F (1,31) = 1.623	p=0.2131				
Sgk1	FLX	F (1,31) = 9.536	p=0.0045	100±4	182±9 ***	98±5	140±10 $^{\#\#}$
	ARS	F (1,31) = 74.28	p<0.0001				
	FLX X ARS	F (1,31) = 7.695	p=0.0095				
Gadd45b	FLX	F (1,31) = 19.31	p=0.0001	100±6	77±7	127±10 *	110±3
	ARS	F (1,31) = 8.602	p=0.0066				
	FLX X ARS	F (1,31) = 0.1691	p=0.6841				
Total *Bdnf*	FLX	F (1,31) = 85.6	p<0.0001	100± 10	137±12 *	204±9 ***	206±6
	ARS	F (1,31) = 4.46	p=0.0437				
	FLX X ARS	F (1,31) = 3.65	p=0.437				
Bdnf long 3' UTR	FLX	F (1,31) = 8.81	p=0.0061	100±3	83±5	106±5 *	101±4
	ARS	F (1,31) = 7.13	p=0.0125				
	FLX X ARS	F (1,31) = 2.01	p=0.1676				
Bdnf IV	FLX	F (1,31) = 17.00	p=0.0003	100±4	124±6	134±8 **	143±7
	ARS	F (1,31) = 6.472	p=0.0168				
	FLX X ARS	F (1,31) = 1.581	p=0.2190				
Bdnf VI	FLX	F (1,31) = 2.330	p=0.1381	100±8	80±4 *	96±3	99±5
	ARS	F (1,31) = 2.633	p=0.1159				

	FLX X ARS	F (1,31) = 5.160	p=0.0310				

Supplementary Table 8: Statistical analysis of *Arc*, *cFos, Zif268*, *Nr4a1*, *Npas4, Dusp1, Sgk1*, *Gadd45β β*, total *Bdnf*, *Bdnf* long 3'UTR, *Bdnf* isoform IV *Bdnf* isoform VI mRNA levels in the **dorsal hippocampus of female rats** exposed to postnatal FLX and subjected to acute restraint stress (ARS) during adolescence. Data are expressed as mean ± SEM of independent measures. *p<0.05, **p<0.01, ***p<0.001 vs vehicle no stress; ##p<0.01 vs postnatal-FLX no stress; two-way ANOVA with Tukey's multiple comparison test.

Gene	Two-way ANOVA	F (dFn, dFd)	p value	Tukey's multiple comparisons test			
				Vehicle no stress	Vehicle ARS	postnatal-FLX no stress	postnatal-FLX ARS
Arc	FLX	F (1,31) = 0.2737	p=0.1301	100±10	137±18	113±14	110±9
	ARS	F (1,31) = 1.667	p=0.2073				
	FLX X ARS	F (1,31) = 2.433	p=0.1301				
cFos	FLX	F (1,29) = 1.210	p=0.2813	100±14	233±49	137±30	298±65
	ARS	F (1,29) = 9.985	p=0.0040				
	FLX X ARS	F (1,29) = 0.09121	p=0.7651				
Zif268	FLX	F (1,31) = 10.64	p=0.0029	100±6	115±17	138±10	151±10
	ARS	F (1,31) = 1.628	p=0.2125				
	FLX X ARS	F (1,31) = 0.007876	p=0.9299				
Nr4a1	FLX	F (1,31) = 0.4406	p=0.5122	100±8	96±15	75±11	135±8
	ARS	F (1,31) = 6.744	p=0.0148				
	FLX X ARS	F (1,31) = 9.098	p=0.0054				
Npas4	FLX	F (1,30) = 1.205	p=0.2821	100±10	96±12	131±35	110±11
	ARS	F (1,30) = 0.3652	p=0.5507				
	FLX X ARS	F (1,30) = 0.1892	p=0.6670				
Dusp1	FLX	F (1,30) = 2.823	p=0.1044	100±9	150±11 *	124±6	162±15
	ARS	F (1,30) = 16.47	p=0.0004				
	FLX X ARS	F (1,30) = 0.2713	p=0.6067				
Sgk1	FLX	F (1,31) = 6.192	p=0.0191	100±4	191±7 ***	115±3	201±6 ###
	ARS	F (1,31) = 287.0	p<0.0001				
	FLX X ARS	F (1,31) = 0.1465	p=0.7047				
Gadd45b	FLX	F (1,31) = 2.577	p=0.1197	100±8	133±9 *	127±5 *	129±6
	ARS	F (1,31) = 5.970	p=0.0211				
	FLX X ARS	F (1,31) = 5.376	p=0.0279				
Total *Bdnf*	FLX	F (1,31) = 55.3	p<0.0001	100± 6	72±7	135±11 *	157±7
	ARS	F (1,31) = 0.122	p=0.7292				
	FLX X ARS	F (1,31) = 9.52	p<0.0045				
Bdnf long 3' UTR	FLX	F (1,31) = 2.831	p=0.1036	100±6	107±7	129±8 *	99±3 #
	ARS	F (1,31) = 3.238	p=0.0828				
	FLX X ARS	F (1,31) = 18.457	p=0.0070				
Bdnf IV	FLX	F (1,31) = 0.5595	p=0.4607	100±6	97±6	101±9	106±2
	ARS	F (1,31) = 0.04317	p=0.8369				
	FLX X ARS	F (1,31) = 0.4149	p=0.5247				
Bdnf VI	FLX	F (1,31) = 1.076	p=0.3085	100±5	77±4 **	92±4	76±4
	ARS	F (1,31) = 18.98	p=0.0002				

	FLX X ARS	F (1,31) = 0.5786	p=0.4532

Supplementary Table 9: Statistical analysis of *Arc*, *cFos, Zif268*, *Nr4a1*, *Npas4, Dusp1, Sgk1*, *Gadd45β β*, total *Bdnf*, *Bdnf* long 3'UTR, *Bdnf* isoform IV *Bdnf* isoform VI mRNA levels in the **dorsal hippocampus of male** rats exposed to postnatal FLX and subjected to acute restraint stress (ARS) during adolescence. Data are expressed as mean ± SEM of independent measures. *p<0.05, **p<0.01, ***p<0.001 vs vehicle no stress; #p<0.05, ##p<0.01, ###p<0.001 vs postnatal-FLX no stress; two-way ANOVA with Tukey's multiple comparison test.

Gene	Two-way ANOVA	F (dFn, dFd)	p value	Tukey's multiple comparisons test			
				Vehicle no stress	Vehicle ARS	postnatal-FLX no stress	postnatal-FLX ARS
Arc	FLX	F (1,30) = 3.416	p=0.0756	100±12	166±15	127±16	203±25 #
	ARS	F (1,30) = 16.50	p=0.0004				
	FLX X ARS	F (1,30) = 0.07230	p=0.7901				
cFos	FLX	F (1,31) = 0.5771	p=0.4538	100±17	614±50 ***	178±50	623±88 ###
	ARS	F (1,31) = 70.67	p<0.0001				
	FLX X ARS	F (1,31) = 0.3701	p=0.5478				
Zif268	FLX	F (1,30) = 28.58	p<0.0001	100±8	184±7 ***	193±22 ***	234±8
	ARS	F (1,30) =21.83	p<0.0001				
	FLX X ARS	F (1,30) = 2.668	p=0.1140				
Nr4a1	FLX	F (1,31) = 0.005830	p=0.9397	100±9	165±18 **	115±7	148±8
	ARS	F (1,31) = 18.38	p=0.0002				
	FLX X ARS	F (1,31) = 1.919	p=0.1769				
Npas4	FLX	F (1,31) = 2.645	p=0.1151	100±10	72±13	51±15	69±16
	ARS	F (1,31) = 0.09790	p=0.7567				
	FLX X ARS	F (1,31) = 1.923	p=0.1765				
Dusp1	FLX	F (1,31) = 4.474	p=0.0434	100±6	108±5	83±9	98±5
	ARS	F (1,31) = 3.193	p=0.0848				
	FLX X ARS	F (1,31) = 0.3471	p=0.5605				
Sgk1	FLX	F (1,31) = 7.053	p=0.0129	100±8	180±13 ***	100±7	131±8
	ARS	F (1,31) = 36.09	p<0.0001				
	FLX X ARS	F (1,31) = 6.769	p=0.0147				
Gadd45β	FLX	F (1,31) = 0.0787	p=0.0581	100±4	126±11	103±9	118±15
	ARS	F (1,31) = 3.90	p=0.0581				
	FLX X ARS	F (1,31) = 0.308	p=0.5832				
Total *Bdnf*	FLX	F (1,31) = 4.264	p=0.0483	100±7	102±5	122±8	111±9
	ARS	F (1,31) = 0.4510	p=0.5074				
	FLX X ARS	F (1,31) = 0.7331	p=0.3991				
Bdnf long 3' UTR	FLX	F (1,31) = 10.5	p=0.0031	100±6	115±3	128±6 *	134±11
	ARS	F (1,31) = 2.19	p=1503				
	FLX X ARS	F (1,31) = 0.405	p=0.5297				
Bdnf IV	FLX	F (1,30) = 3.031	p=0.0931	100±8	134±5 **	109±7	148±6 ##
	ARS	F (1,30) = 30.63	p<0.0001				
	FLX X ARS	F (1,30) = 0.1819	p=0.6731				
Bdnf VI	FLX	F (1,30) = 1.401	p=0.0465	100±5	83±4	95±4	100±7
	ARS	F (1,30) = 1.342	p=0.2565				

	FLX X ARS	F (1,30) = 5.368	p=0.0280

Supplementary Table 10: Statistical analysis of *Arc*, *cFos, Zif268*, *Nr4a1*, *Npas4, Dusp1, Sgk1*, *Gadd45β β*, total *Bdnf*, *Bdnf* long 3'UTR, *Bdnf* isoform IV *Bdnf* isoform VI mRNA levels in the **ventral hippocampus of female** rats exposed to postnatal FLX and subjected to acute restraint stress (ARS) during adolescence. Data are expressed as mean ± SEM of independent measures. *p<0.5, **p<0.01, ***p<0.001 vs vehicle no stress; #p<0.05, ##p<0.01, ###p<0.001 vs postnatal-FLX no stress; two-way ANOVA with Tukey's multiple comparison test.

Gene	Two-way ANOVA	F (dFn, dFd)	p value	Tukey's multiple comparisons test			
				Vehicle no stress	Vehicle ARS	postnatal-FLX no stress	postnatal-FLX ARS
Arc	FLX	F (1,29) = 0.01046	p=0.9193	100±14	150±17	142±20	112±12
	ARS	F (1,29) = 0.3880	p=0.5388				
	FLX X ARS	F (1,29) = 6.151	p=0.0199				
cFos	FLX	F (1,31) = 4.183	p=0.0503	100±37	581±93	768±301	638±158
	ARS	F (1,31) = 0.9773	p=0.3313				
	FLX X ARS	F (1,31) = 2.971	p=0.0958				
Zif268	FLX	F (1,30) = 60.22	p<0.0001	100±6	115±8	186±13 ***	180±11
	ARS	F (1,30) = 0.1901	p=0.6663				
	FLX X ARS	F (1,30) = 1.195	p=0.2840				
Nr4a1	FLX	F (1,30) = 18.80	p=0.0002	100±9	127±8	167±24 *	190±12
	ARS	F (1,30) = 2.705	p=0.1116				
	FLX X ARS	F (1,30) = 0.01994	p=0.8888				
Npas4	FLX	F (1,30) = 7.063	p=0.0131	100±17	54±7	199±59	141±31
	ARS	F (1,30) = 2.179	p=0.1515				
	FLX X ARS	F (1,30) = 0.03036	p=0.8630				
Dusp1	FLX	F (1,30) = 0.4520	p=0.5071	100±5	139±7 **	139±11 **	110±2
	ARS	F (1,30) = 0.4091	p=0.5278				
	FLX X ARS	F (1,30) = 19.38	p=0.0002				
Sgk1	FLX	F (1,31) = 1.348	p=0.2553	100±7	141±7 **	91±5	168±10 ###
	ARS	F (1,31) = 59.55	p<0.0001				
	FLX X ARS	F (1,31) = 5.280	p=0.0293				
Gadd45β	FLX	F (1,30) = 3.60	p=0.0684	100±9	153±10 *	146±13	156±16
	ARS	F (1,30) = 5.92	p=0.0219				
	FLX X ARS	F (1,30) = 2.84	p=0.1036				
Total *Bdnf*	FLX	F (1,30) = 10.79	p=0.0028	100±5	89±7	11±7	120±6
	ARS	F (1,30) = 0.02828	p=0.8677				
	FLX X ARS	F (1,30) = 2.428	p=0.1308				
Bdnf long 3' UTR	FLX	F (1,30) = 2.415	p=0.1318	100±4	119±3 *	126±6 **	107±3 #
	ARS	F (1,30) = 0.01729	p=0.8964				
	FLX X ARS	F (1,30) = 17.57	p=0.0003				
Bdnf IV	FLX	F (1,30) = 4.132	p=0.0520	100±3	124±4	129±12 *	124±4
	ARS	F (1,30) = 1.606	p=0.2158				
	FLX X ARS	F (1,30) = 4.082	p=0.0534				
Bdnf VI	FLX	F (1,30) = 4.988	p=0.0340	100±4	88±3	113±5	94±5 #

ARS	F (1,30) = 13.78	p=0.0009
FLX X ARS	F (1,30) = 0.7708	p=0.3877

Supplementary Table 11: Statistical analysis of *Arc*, *cFos, Zif268*, *Nr4a1*, *Npas4, Dusp1, Sgk1*, *Gadd45β* mRNA levels in the **ventral hippocampus of male** rats exposed to postnatal FLX and subjected to acute restraint stress (ARS) during adolescence. Data are expressed as mean ± SEM of independent measures. *p<0.05, **p<0.01, ***p<0.001 vs vehicle no stress; #p<0.05, ###p<0.001 vs postnatal-FLX no stress; two-way ANOVA with Tukey's multiple comparison test.

Gene	Forward Primer	Reverse Primer	Probe
Arc	GGTGGGTGGCTCTGAAGAAT	ACTCCACCCAGTTCTTCACC	GATCCAGAACCACATGAATGGG
c-Fos	TCCTTACGGACTCCCCAC	CTCCGTTTCTCTTCCTCTTCAG	TGCTCTACTTTGCCCCTTCTGCC
Zif268	GAGCGAACAACCCTACGAG	GTATAGGTGATGGGAGGCAAC	TCTGAATAACGAGAAGGCGCTGGTG
Npas4	TCATTGACCCTGCTGACCAT	AAGCACCAGTTTGTTGCCTG	TGATCGCCTTTTCCGTTGTC
Dusp1	TGTGCCTGACAGTGCAGAAT	ATCTTTCCGGGAAGCATGGT	ATCCTGTCCTTCCTGTACCT
Sgk1	GACTACATTAATGGCGGAGAGC	AGGGAGTGCAGATAACCCAAG	TGCTCGCTTCTACGCAGC
**Total *Bdnf***	AAGTCTGCATTACATTCCTCGA	GTTTTCTGAAAGAGGGACAGTTTAT	TGTGGTTTGTTGCCGTTGCCAAG
36b4	TCAGTGCCTCACTCCATCAT	AGGAAGGCCTTGACCTTTTC	TGGATACAAAAGGGTCCTGG

Supplementary Table 12: Sequences of forward and reverse primers and probes used in real-time PCR analyses and purchased form Eurofins MWG-Operon.

Gene	Accession number	Assay ID
Nr4a1	BC097313.1	Rn01533237_m1
Gadd45β	BC085337.1	Rn01452530_g1
***Bdnf* long 3'UTR**	EF125675	Rn02531967_s1
***Bdnf* isoform IV**	EF125679	Rn01484927_m1
***Bdnf* isoform VI**	EF125680	Rn01484928_m1

Supplementary Table 13. Probes purchased form Life Technologies which did not disclose the sequence.
